# Intramuscular Epinephrine-Induced Transient ST-Elevation Myocardial Infarction

**DOI:** 10.1177/2324709618785651

**Published:** 2018-07-06

**Authors:** Binav Shrestha, Paritosh Kafle, Shivani Thapa, Suyash Dahal, Vijay Gayam, Alix Dufresne

**Affiliations:** 1 Interfaith Medical Center, Brooklyn, NY, USA; 2 KIST Medical College and Teaching Hospital, Lalitpur, Kathmandu, Nepal

**Keywords:** anaphylaxis, STEMI, epinephrine, Kounis syndrome, transient

## Abstract

*Introduction.* Myocardial infarction in the setting of anaphylaxis may result from the anaphylaxis itself or from the epinephrine used to treat the anaphylaxis. While cases of myocardial infarction due to large doses of intravenous epinephrine have previously been reported, myocardial infarction after therapeutic doses of intramuscular epinephrine is rarely reported. *Case Report.* A 23-year-old male presented with sudden onset of difficulty in swallowing and speech after eating takeout food. He was treated with intramuscular epinephrine for presumed angioedema following which he immediately developed chest tightness associated with ST elevation on electrocardiogram and elevated serum troponin. His symptoms and electrocardiogram findings were transient and resolved within the next 10 minutes. *Conclusion*. Epinephrine is lifesaving during anaphylaxis and should be promptly used. Health care providers, however, need to be aware and vigilant of this rare complication of epinephrine.

## Introduction

Anaphylaxis is a severe, systemic allergic reaction and is characterized by involvement of different body systems. Myocardial infarction (MI) in the setting of anaphylaxis has also been reported before.^1,2^ It may result from the anaphylaxis itself or from the epinephrine used to treat the anaphylaxis.^1-6^ While cases of MI due to large doses of intravenous epinephrine have previously been reported, MI after therapeutic doses of intramuscular epinephrine is rarely reported.^7-10^ In this article, we present a case of acute MI in a young healthy patient after the use of intramuscular epinephrine for presumed anaphylaxis and review the literature for similar cases.

## Case Report

**Figure 1.**
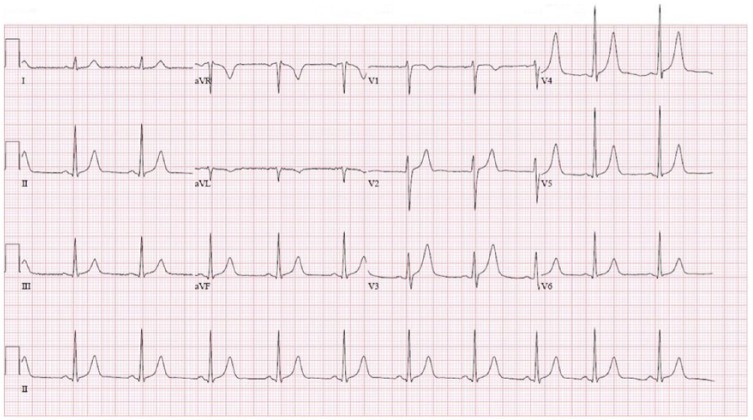
Electrocardiogram at presentation.

A 23-year-old male came to the emergency department with excessive salivation, difficulty in swallowing, and speech immediately after eating some Chinese food. It was associated with retching but no vomiting, abdominal pain, diarrhea, chest pain, cough, or difficulty in breathing. Rest of the review of symptoms was negative. He had no significant past medical history. However, he reported multiple similar episodes in the past for which he had undergone extensive gastroenterological workup, the details of which he did not know at presentation. He was a nonsmoker and did not use any drugs. He had no known drug or food allergy and reported eating the same food from the same eatery multiple times in the past with no reaction. At presentation, his temperature was 98.2°F, pulse was 73 beats per minute, blood pressure was 117/42 mm Hg, and respiratory rate was 22 breaths per minute with oxygen saturation of 97% in room air. Physical examinations were significant for drooling and hyperventilation with diffuse wheezing on chest auscultation. Complete blood counts revealed hemoglobin of 15 g/dL (normal 13-17 g/dL), hematocrit of 45% (normal 39% to 53%), platelet count of 234 000/µL (normal 130 000-400 000/µL), and a white cell count of 13 000/µL (normal 4500-11 000/µL) with a neutrophil of 47% (normal 40% to 70%), lymphocyte of 42% (normal 22% to 48%), eosinophil of 2.6% (normal 0.5% to 5%), and basophil of 0.6% (normal 0% to 2%). Rest of the blood works including blood chemistry and coagulation profile was normal. Chest X-ray was normal. The initial troponin was 0.01 ng/mL, and the electrocardiogram showed normal sinus rhythm with no acute ST or T wave changes (Figure 1). Urine toxicology screen was negative.

**Figure 2.**
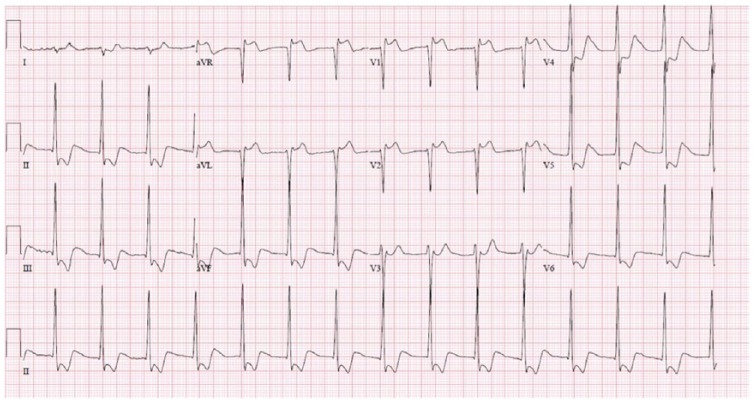
Electrocardiogram immediately after epinephrine administration.

**Figure 3.**
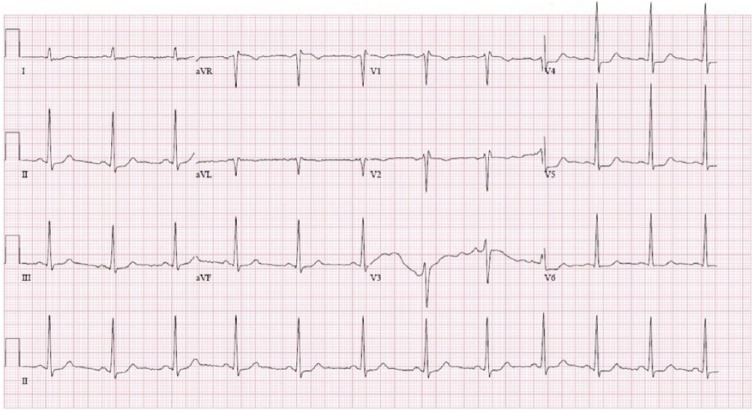
Electrocardiogram (EKG) after 10 minutes of the second EKG.

So with the presumed diagnosis of angioedema, he was treated with 125 mg of intravenous methylprednisolone, 50 mg of intramuscular diphenhydramine, and 0.5 mg of intramuscular epinephrine. However, immediately after the administration of epinephrine, he started to complain of palpitation and increasing chest tightness. His pulse jumped to around 150 beats per minute, while his blood pressure dropped to 98/64 mm Hg. Repeat electrocardiogram (EKG) showed ST elevation in V1, V2, and aVR, and ST depression in leads II, III, aVF, and V4-V6 (Figure 2). Serum troponin jumped to 2.14 ng/mL. He was treated with 1 inch of topical nitroglycerin paste, 325 mg of aspirin, 40 mg of atorvastatin, and intravenous normal saline. His chest pain and palpitation resolved over the next 10 minutes with resolution of his EKG changes (Figure 3). Echocardiography was done subsequently and showed normal systolic and diastolic function with no regional wall abnormality. Over the course of the next day, his serum troponin rose to 2.30 ng/mL before trending downward to 1.3 ng/mL, and his presenting symptoms of difficulty in swallowing and speech resolved. However, given his low cardiac risk factors and the likely cause for the myocardial ischemia being coronary artery vasospasm rather than atherosclerotic disease, cardiac angiography was not done. During the course of his hospitalization, records of his previous gastroenterological workup were obtained that showed severe tightening of proximal esophageal sphincter with distal esophagitis on enterogastroduodenoscopy and normal gatroesophageal peristalsis on radiocontrast study. In the background of these findings with recurrent similar symptoms in the past and absence of other findings suggestive of allergy, his presenting symptoms were attributed more to his esophageal spasm and less to an allergic reaction. The patient was then discharged home to follow-up as an outpatient.

## Discussion

Anaphylaxis is a severe, systemic allergic reaction and is characterized by involvement of different body systems. Acute MI during anaphylactic reaction has been previously reported and may result from the anaphylaxis itself or from the epinephrine used to treat the anaphylaxis.^1-4^ Kounis syndrome is the nonthrombogenic cause of myocardial ischemia or infarction triggered by the release of inflammatory mediators during an allergic or anaphylactic reaction.^11^ The main pathophysiological pathway is thought to be the activation and degranulation of mast cells within the coronary arteries during these allergic reactions with release of chemical mediators like histamine and tryptase resulting in coronary vasospasm.^12,13^ Epinephrine used for the treatment of an anaphylactic reaction has also been rarely reported as an independent cause for acute MI during anaphylaxis.^3-6^ Like in our case, the temporal relationship between epinephrine administration and development of acute MI favors epinephrine as the cause of MI in all these cases rather than Kounis syndrome. Furthermore, though initially treated with epinephrine for presumed angioedema, our patient’s presentations occurred in the absence of any identifiable trigger for angioedema and were more likely due to his long-standing paroxysmal esophageal spasms and not any allergic reaction. This lack of allergic milieu further supports epinephrine more than Kounis syndrome as the cause for the development of acute MI in our patient.

Epinephrine is the drug of choice in anaphylactic reactions. The β-adrenergic properties of epinephrine cause bronchodilation, increase myocardial output and contractility, and suppress further mediator release from mast cells and basophils, while the α-adrenergic properties cause peripheral vasoconstriction. It normally increases the coronary blood flow as its indirect vasodilatory effects—increased duration of diastole, elevated mean arterial pressure, and increased metabolic demand of the myocardium—offset the direct vasoconstrictive effect on the coronary arteries. While the exact mechanism has not been elucidated, development of MI with epinephrine involves imbalance between its direct vasoconstrictive and indirect vasodilatory effects on the coronary arteries. Additionally, epinephrine may potentiate platelet aggregation by increasing adenosine diphosphate and arachidonate-induced thromboxane B2 production.^14^ Most cases of MI with epinephrine use have been reported in patients who received high intravenous doses of epinephrine.^8-10^ Cases of MI with low doses or other routes of epinephrine as in our case, however, are rare, with our review of literature yielding only 7 other cases (Table 1). Most of these patients were young with a median age of 42 years and had no or only few cardiac risk factors. They received low therapeutic doses of epinephrine for an allergic or anaphylactic reaction. Route of administration was not a predetermining factor for the development of MI as it was seen with intravenous, intramuscular, as well as subcutaneous routes. These patients immediately developed chest pain after receiving epinephrine with the median time between administration of epinephrine and development of symptoms being 5 minutes. Their symptoms were accompanied by acute ST-segment changes on EKG and rise in serum troponin. The symptoms and EKG changes, however, were transient and lasted less than 30 minutes in most cases. And among the 6 cases that underwent coronary angiogram, all had patent coronary arteries except for the 1 case described by Tummala et al.^17^ Absence of typical cardiac risk factors and presence of patent coronaries in most of these cases suggest a vasospastic mechanism for MI. Identifying patients at risk of developing coronary artery vasospasm following administration of therapeutic doses of epinephrine needs further exploring.

**Table 1.** List of Published Cases of Myocardial Infarction Following Administration of Epinephrine.

Author	Age of Patient	Sex of Patient	Number of Typical Cardiac Risk Factors	Dose of Epinephrine (mg)	Route of Epinephrine	Symptoms	Time to Onset of Symptoms (Minutes)	Duration of Symptoms (Minutes)	EKG Findings	Troponin Level (ng/mL)	Status of Coronary Arteries
Our case	23	Male	0	0.5	IM	Chest pain and palpitation	5	10	ST-elevation in V1, V2, and aVR with ST-depression in leads II, III, aVF, and V4-V6	2.14	—
Jayamali et al^3^	21	Male	0	0.5	IM	Chest pain and palpitation	10	30	ST-depression	2.15	Patent
Shaver et al^4^	29	Female	2	0.1	IV	Chest pain	<10	45	ST-elevation in leads I, aVL, V2, and V5-6 with ST-depression in III, aVF, and V1	1.99	—
Cunnington et al^5^	43	Female	0	0.5^a^	IM	Chest pain	5	30	ST-depression in V1-V5	0.53	Patent
Goldhaber-Fiebert et al^6^	55	Female	2	0.1	IV	Chest pain	<5	15	ST-elevation in II, III, and aVF	0.23	Patent
Ferry et al^15^	43	Male	0	0.3^a^	SC	Chest pain	<15	—	ST-elevation in aVL, V1-V4 with ST-depression in II, III, aVF, and V5-V6	—	Patent
Caballero et al^16^	41	Male	2	0.5	SC	Chest pain	<5	30	ST-elevation in II, II, and aVF	—	Patent
Tummala et al^17^	62	Male	2	0.5^a^	IM	Chest pain	5	—	ST-elevation in V1-V4 with ST-depression in leads II, III, aVF	0.3	Thrombus in the mid-LAD artery

Abbreviations: EKG, electrocardiogram; IM, intramuscular; IV, intravascular; LAD, left anterior descending; SC, subcutaneous.

a Patient received a total of 2 doses.

Despite these risk of MI on rare occasions, epinephrine is lifesaving during anaphylaxis and should be promptly used as the risks of anaphylaxis far outweigh the risks of the adverse events. However, health care providers need to be aware and vigilant of this rare but potentially catastrophic complication of epinephrine.
